# Is There an “Expert” Stranger Rapist?

**DOI:** 10.1177/1079063221993478

**Published:** 2021-02-15

**Authors:** Julien Chopin, Sarah Paquette, Eric Beauregard

**Affiliations:** 1 Simon Fraser University, Burnaby, British Columbia, Canada; 2 Laval University, Quebec City, Quebec, Canada

**Keywords:** criminal expertise, sexual crime, rational choice theory, modus operandi, forensic awareness strategies, stranger rape

## Abstract

The concept of expertise applied to the criminal context assumes that offenders are driven by the abilities to both maximize the payoffs and minimize the risks associated with the crime-commission. This study tested the articulation between these two types of decisions taken by stranger rapists to successfully commit their crime. Specifically, this study aims to identify whether offenders whose modus operandi is indicative of criminal expertise are more likely to use forensic awareness strategies. Multivariate analyses conducted on 1,551 cases showed that stranger rapists who adopted behaviors indicative of expertise were more likely to use forensic awareness strategies to decrease the risk of police detection. Mixed associations were found between the number of forensic awareness strategies and their nature (i.e., protecting identity vs. destroying evidence) and rapists’ expertise, thus leading to a four-type theoretical classification of expertise: novice, bold, opportunistic, and expert stranger rapists. Implications for research and practice are discussed.

## Introduction

Hirschi (1986) challenged the idea that offending requires sophistication or specialized skills. This notion is especially pertinent to sexual offenders. Although empirical evidence may suggest otherwise (Lussier, Proulx, & LeBlanc, 2005), long-standing beliefs that sexual offenders are driven by an uncontrollable sexual urge remain in conventional wisdom (e.g., Simon et al., 2000). In fact, findings suggest quite the contrary, as sexual offenders are much more similar to other nonsexual offenders than previously thought (e.g., Harris et al., 2009; Lussier & Healey, 2009; Lussier, LeBlanc, & Proulx, 2005). For instance, previous studies focusing on offense chain models suggested that there exists several patterns of sexual offending influenced by positive or negative background factors (e.g., lifestyle, personal circumstances) and goals (e.g., seeking sexual gratification to enhance positive or negative mood; see, for example, Gannon et al., 2010; Polaschek et al., 2001; Polaschek & Hudson, 2004; Ward et al., 2006).

Accordingly, an important criminological question with theoretical and practical implications pertains to whether offending requires specialized skills and sophistication. On one hand, it has been argued that successfully engaging in criminality does not require special skills, as evidenced by the lack of premeditation involved in most crimes (see Beauregard & Proulx, 2017). On the other hand, it has been argued that this apparent absence in decision-making is not an indication of lack of skills and planning, but rather it demonstrates that some offenders have developed in-depth knowledge and skills to assess various situations and opportunities and therefore have become better and quicker to act on them (Nee, 2015). This latter hypothesis has found support within a large body of research on the role of expertise in decision-making in fields unrelated to crime (e.g., chess, medicine, sports, and aviation; see Nee & Ward, 2015). Research on criminal expertise in the context of sexual offending has both theoretical and practical import. First, the study of criminal expertise could provide a better understanding of the issue of repetition in sexual offending. An expert offender should be more likely to be persistent and to repeat the same type of crime than other offenders (see, for example, Swartout et al., 2015). Second, as noted by Bourke et al. (2012), the identification of criminal expertise would allow to better understand why some clinical and rehabilitation programs do not work for some individuals involved in sexual crimes (i.e., use of specific expert skills to block or delay treatment).

The analysis of criminal expertise can be made through both structural (i.e., cognitive schemas and skills) and behavioral representations (i.e., expertise in modus operandi and offense behavior; Nee & Ward, 2015). Although Nee and Ward (2015) noted that the analysis of both cognitive and behavioral processes is relevant to identifying patterns of expertise, few studies have focused specifically on crime scene behaviors. In fact, research on behavioral indicators primarily focused on the precrime phase, and little attention has been dedicated to the entire crime-commission process. Nonetheless, research demonstrates that the use of behavioral indicators is especially relevant for analyzing the expertise in sexual offending. As such, these studies highlight the role of an offenders skills and experience in the development of an expertise for offending (Bourke et al., 2012; Ciardha, 2015; Ward, 1999).

As such, the current study adopts an expertise framework to test whether individuals who have committed stranger rapes have criminal expertise using behavioral indicators. This study aims to specifically focus on offenders’ behaviors associated with expertise in sexual offending. More specifically, when analyzing the phases of the crime-commission process (i.e., pre-, peri-, and postcrime), is it possible that some offenders who use a sophisticated modus operandi behavior will also adopt specific strategies that may contribute to avoiding detection?

### Expertise and Decision-Making

Research on notions of expertise has been conducted across various disciplines and contexts—from chess players, pilots, doctors (e.g., Vicente & Wang, 1998) to criminals (e.g., Jacobs, 1996; Nee & Meenaghan, 2006; Topalli, 2005; Wright et al., 1995). Whatever the domain, expertise may be defined as the acquisition of cognitive processes and behaviors, drawn from experience that prove more effective than to those new to a given field (Nee & Ward, 2015). Thus, expert decision-making is characterized by an absence of explicit deliberation, a greater speed of performance, and a capacity to multitask. Conversely, novices need explicit instruction and tend to be slower and easily distracted whenever experiencing new tasks (see Palmeri et al., 2004). As decision-making becomes more automatic and unconscious, this enables a person to free up space for other deliberations and problem-solving tasks (Logan & Etherton, 1994). It has been suggested that, in any given domain—criminal or noncriminal—the achieved level of expertise should be seen as a continuum rather than as a present–absent dichotomy (Nee & Ward, 2015) and is obtained as a result of deliberate practice as opposed to innate ability (e.g., Ericsson, 2006). This is congruent with previous observations suggesting that the skills associated with expertise are context bound and may not necessarily transfer to different domains (see Ericsson & Charness, 1994). Moreover, as highlighted by Ciardha (2015), even experts’ performance may be impaired under certain circumstances, such as cognitive biases (e.g., tunnel vision; see Dror, 2011), errors in judgment (e.g., overestimation of ability in a new situation; Chi, 2006), or risky decision-making (e.g., due to the effects of drug use; Weinborn et al., 2013).

The notion of expertise in crime may be directly linked to rational choice theory (RCT; Cornish & Clarke, 1986, 1987). According to RCT, offenders develop skills to assess and respond to crime opportunities through practice (Nee & Ward, 2015). Thus, while assessing the risks and rewards associated with committing the crime, offenders will make a decision to act in a certain way to reduce the risks and to improve the rewards (Nee & Taylor, 2000). Because crime is viewed as a dynamic process influenced by situational factors, offenders are assumed to improve their decision-making through experience and learn to modify their strategies to commit crimes (Cornish, 1994).

Decisions-making, however rudimentary this process may appear, exhibits a measure of rationality, albeit constrained by limits of time, ability, and the availability of relevant information (Cornish & Clarke, 1986). Although often presented as limited rationality (Simon, 1957), Gigerenzer and Selten (2002) suggested that this “bounded” rationality should not be seen as limited. Instead, they proposed that bounded rationality represents an adaptation allowing to maximize successful outcomes under typical constraints (e.g., time, availability of information). As demonstrated by empirical studies (see Gigerenzer & Selten, 2002, for a review), offenders are more likely to use heuristics to make their decisions instead of a thorough cost–benefit analysis as implied in the traditional RCT. The RCT has been recently applied to sexual offenders, and what these studies have found is that even for sexual crimes, offenders follow a rational decision-making process to achieve their objectives (see, for example, Beauregard et al., 2007; Chopin & Beauregard, 2020a; Pedneault et al., 2017).

### Criminal Expertise and Avoiding Detection

Criminal expertise has often been discussed in relation to burglary (Nee, 2015), and empirical findings have shown that these offenders are capable of discriminating between targets using environmental cues such as occupancy and accessibility (e.g., Bennett & Wright, 1984). Relatedly, Logie et al. (1992) showed that teenage-experienced burglars demonstrated a more efficient and automatic memory for environmental cues compared with other offenders, police officers, and students. Moreover, Nee and colleagues (Nee et al., 2015; Nee & Taylor, 2000; Taylor & Nee, 1988) illustrate/demonstrate/assert that in comparison with noncriminals, burglars used distinctive and systematic routes and relied on previous learning experiences in their decision-making for target selection. Nee and Meenaghan (2006) suggested that burglars developed—through skills and expertise from prior experiences—a rational model of target appraisal based on cues indicative of relative wealth, occupancy, access, and security.

Although the above examination of expertise emphasizes the strategies used by burglars to successfully commit crimes, the notion also applies to expertise that can be further extended to the entire offending population. This is because, at some point, all offenders will have to assess risks associated with getting caught. Research has shown that experienced offenders involved in various types of crimes may develop a set of skills designed to reduce the risks of police detection and accordingly apprehension (e.g., Cherbonneau & Copes, 2005; Copes & Cherbonneau, 2006; Gallupe et al., 2011; Holt et al., 2014).

Interestingly, property crimes have received the most scrutiny in terms of detection avoidance strategies. Although scholars recognize that some offenders present a certain level of expertise regarding their crimes, they have been hesitant to apply this concept to the more violent crimes, almost as if violent criminals were not capable of expertise due to the impulsivity involved in these crimes (see Day & Bowen, 2015). There is, however, empirical evidence showing a part of violent offenders, including those who engage in sexual abuse, are displaying expertise in their offending behaviors.

### Expertise in Sexual Offending

Ward (1999) was the first to apply the concept of expertise to sexual offenders. Without denying the need to look at their deficiencies to provide them with appropriate coping skills, Ward (1999) argued that some offenders demonstrate expertise—or a dysfunctional expertise (Nee & Ward, 2015)—in the commission of sexual crimes. As illustrated by Ciardha (2015), “with sufficient rehearsal and repetition, an individual may become an expert in a particular area, regardless of whether that behavior is prosocial or antisocial. Sexual offending is unlikely to be an exception” (p. 26). For instance, it was suggested that exposure to pornography and networking with other offenders (e.g., online sexual offenders against children) could provide rich knowledge that could then be applied to their behavioral strategies when offending (see Fortin et al., 2018). Moreover, “frequent masturbation to deviant sexual fantasies could arguably provide a form of practice-based or mental rehearsal as outlined by the expertise and expert performance literature” (Bourke et al., 2012, p. 2393). Ward (1999) also highlighted various mechanisms that could contribute to the development of this expertise in sexual offenders, such as learning—observational (i.e., via other offenders) and symbolic modeling (e.g., pornography) as well as through their own sexual abuse experience. Bourke et al. (2012) found that some skills can be acquired during the offender’s early life. They noted that sexualization (i.e., sexualized behaviors from early childhood) and deviant norms and schemas (i.e., maladaptive norms/beliefs dysfunctional thoughts of behaviors) from experiences of early child abuse characterized expert child abusers. They used their own past victimization experience to manipulate and sexually assault children later in life (Bourke et al., 2012).

Specifically related to strategies used to reduce the risks of police detection, Ward (1999) suggested that avoiding police detection among individuals who sexually abuse several victims over many years was the result of a specific expertise, for example, taking precautions with offense locations, being able to regulate their emotional state, deceiving people close to them, and conducting constant risk appraisal that increases their chances to avoid detection. These various strategies are assumed to be incorporated in multiple cognitive scripts about different elements of an offense (Bourke et al., 2012; Ward, 1999). These scripts therefore guide decision-making and the pursuit of objectives that ultimately contribute to better modulate individuals’ own behaviors, thus revealing a certain expertise. For instance, in a study looking at child sexual offenders, Bourke et al. (2012) found empirical support for the existence of a continuum of expertise, from novice to expert. Similarly, Lussier et al. (2011) tested two dimensions of criminal achievement, namely, productivity and cost avoidance. These authors found that offenders who specialize in sexual crimes were more likely to be the most productive and the least detected offenders. Pedneault et al. (2017) investigated the decision-making process of sexual offenders and how their decisions influenced the desired and undesired crime outcomes. Findings show that decisions made by sexual offenders in the context of their crimes were mostly oriented toward the production of immediate positive outcomes (e.g., crime completion, sexual penetration) and the prevention of immediate negative (e.g., verbal resistance, nonviolent physical resistance) outcomes. Results therefore suggest that sexual offenders demonstrated little consideration for nonimmediate negative outcomes, which could include, for example, a future apprehension by the police.

Subsequent studies focusing on criminal expertise primarily relied on convicted offenders in their samples. As such, this body of literature demonstrates an association between past criminal experience and actual offending expertise, thereby suggesting that sexual offenders have learned from their previous experiences and attempt not to repeat the same mistakes (Chopin, Beauregard, & Bitzer, 2020; Lussier et al., 2011; Lussier & Mathesius, 2012), and such conceptualization is incomplete. Indeed, additionally or complementary with past criminal experience, other ways are also presumed to contribute to the acquirement of an expertise (e.g., watching pornography, networking with other offenders). Therefore, the analysis of expertise should not solely focus on experienced offenders but should include offenders who may have been skilled in other ways.

### Forensic Awareness and Avoiding Detection in Sexual Crimes

Forensic awareness is one specific related aspect of detection avoidance strategies that has developed in parallel of the concept of expertise. This notion refers to one’s adaptation of his criminal behavioral pattern (i.e., modus operandi) based on situational factors and the use of extra precautions to avoid apprehension (Davies, 1992). Adopting these behaviors and awareness therefore is presumed to be indicative of an understanding of the forensic evidence gathered by police investigators at a crime scene to solve the crime (Beauregard & Martineau, 2014). Only a few studies have investigated detection avoidance strategies among men who had committed sexual crimes against adult victims and fewer had the specific objective of thoroughly analyzing forensic awareness strategies in rape cases. Davies and Dale (1995) found, among a sample of 75 men who had committed rape against strangers, that targeting a victim located in a remote area was indicative of forensic awareness. Examining a sample of 210 cases of sexual abuse against strangers, Davies et al. (1997) identified strategies such as the destruction of semen and precautions to avoid leaving fingerprints on the crime scene. In addition, the authors noted that these strategies may be reflective of the expertise gained over time as men who engaged in such behaviors were more likely to have a criminal record. The extent of the criminal history was also found to be reflective of forensic awareness in another sample of 44 men with serial and nonserial rape offenses (Park et al., 2008). To date, the most detailed study on forensic awareness in men with sexual offenses against adults was conducted by Beauregard and Bouchard (2010). Using a sample of 72 men who had committed 222 rape events, the authors examined whether situational correlates—disinhibitors (e.g., alcohol, drug consumption), specificity in target selection, and evidence left at the crime scene—could predict forensic awareness. Their descriptive analysis showed a variety of strategies more or less frequently used to avoid detection: concealing his identity (44%), preventing his face from being seen (33%), wearing gloves (15%), wiping semen (7%), wearing a condom (2%), lying about his name (1%), making victim shave or shower (1%), and avoiding ejaculation (0.5%). In lights of their findings, Beauregard and Bouchard (2010) concluded that men who offend may be more forensically aware in their preparation, compared with after offending, as many planned to protect their identity but very few got rid of DNA evidence. Furthermore, the results reveal that men under the influence of drugs or alcohol were less forensically aware. On the contrary, forensic awareness was predicted by situational variables such as targeting specific victims (i.e., those alone) and engaging in behaviors that have the possibility of leaving physical evidence (i.e., breaking and entering, penetrating the victim, and ejaculating). Among a sample of 350 cases of sexual homicide, Beauregard and Martineau (2014) examined 16 detection avoidance strategies employed by men who have murdered their victim during a sexual crime; some being more frequent (e.g., body concealed: 36%, body removal: 34%, destruction of the evidence: 31%) than others (e.g., staging the crime scene: 9%, body dismembered: 6.3%). In addition, they found that the use of certain strategies (e.g., removing semen from the scene) over others (e.g., body dismembered) were positively associated with the likelihood that the case would get solved. Finally, Chopin et al. (2019) showed that forensic awareness strategies used by individuals who have committed stranger rapes are not essential to avoid police detection as the solving of the crime was mainly influenced by the modus operandi used by the offender to commit the crime. They also found that some of the forensic awareness strategies actually increased the likelihood of the crime being solved—this was found to be the case for sexual homicide (Beauregard & Martineau, 2014).

### The Use of Forensic Awareness Strategies and the Crime Outcome in Sexual Crimes

It is important to distinguish the ability to use specific skills and adapt one’s behavior to achieve an objective and the actual outcome of that objective. For instance, studies have found a weak association between the use of avoidance strategies by sexual offenders and crime solving (Beauregard & Martineau, 2014; Chopin et al., 2019). Avoiding police detection in sexual crimes is rather more strongly associated with the crime context (Chiu & Leclerc, 2020; Chopin, Beauregard, & Deslauriers-Varin, 2020; Chopin et al., 2019), thwarted investigations (James & Beauregard, 2020), and luck (Balemba et al., 2014; Rossmo, 2009). Therefore, the knowledge and ability to avoid police detection should not be limited to the actual outcome of the crime (i.e., solved/unsolved). Otherwise, the study of criminal expertise would be almost impossible.

## Aim of the Study

Previous empirical studies have shown that contrary to Hirschi’s (1986) view of criminal behavior, some offenders may have expertise related to their specific criminal activity. Although most of these studies have focused on property crimes, some scholars have suggested that sexual offenders may also demonstrate various levels of expertise when committing their crimes. In most studies, expertise has been mainly considered as a cognitive process, neglecting to examine the corresponding behaviors that may have been adopted during the crime-commission process. According to the rational choice perspective, it can be assumed that expert offenders are those adopting behaviors to maximize the payoffs and reduce the risks associated with their crime. More precisely, the maximization of payoffs may be linked to the use of a sophisticated modus operandi (i.e., set of acts and decisions allowing offenders to successfully commit their crimes), whereas the minimization of risks may be associated with the police avoidance strategies used by offenders. Based on these two axes, this exploratory study proposes to focus on a new approach to investigate the issue of criminal expertise: the study of behavioral indicators of expertise rather than cognitive processes. We formulate the general hypothesis that expert individuals who have committed stranger rapes are those adopting both a sophisticated modus operandi and strategies to avoid police detection. Therefore, using a large sample of stranger rape cases involving adult victims, this study aims to identify (a) whether offenders whose modus operandi is indicative of criminal expertise are more likely to use forensic awareness strategies, and (b) whether modus operandi behaviors most related to criminal expertise are also positively related to more sophisticated types of forensic awareness strategies.

## Method

### Sample

We used a sample of 1,551 solved rape cases that occurred in France (metropolitan and overseas) between 2001 and 2018. Specifically, 64.99% (*n* = 1,008) of the cases occurred between 2001 and 2010, and 35.01% (*n* = 543) of the cases occurred between 2010 and 2018. Although all cases have been solved by the police—hence all offenders have been caught at some point—the current study focuses on the sophistication and expertise involved in stranger rapists’ crime-commission process, not the actual outcome of this process.

The sample was taken from a national police database operated by the Ministry of Interior in France. This database is maintained by crime analysts using different sources of information relating to criminal cases. Detailed and unique information on the crime-commission process comes from investigation files that are completed by criminal investigators as well as offenders’ and victims’ interviews, whereas forensic awareness strategies information come from forensic services, legal medicine, and interviews with victims. All this information is compiled, analyzed, and entered in the database by a team of crime analysts, experts in violent crimes. Crime analysts are also familiar with how each variable is operationalized. Although it is still possible to encounter missing values as information may not always be known by the investigators, this was not the case with the variables examined in the current study.

According to previous studies in the field, we made several methodological choices. First, rape cases included in the current sample were perpetrated by offenders both with and without previous criminal experiences. This is an advantage over past studies that have only considered offenders with past criminal experience as expertise can be learn from various sources. Second, cases included in our study had to present either vaginal or anal penetration on a victim aged 16 years or older. Several studies used this cutoff to discriminate child abuses from adults’ sexual assaults as there are major differences between the modus operandi used to assault children and adult victims (see Chopin, 2017; Chopin & Beauregard, 2019b; Chopin & Caneppele, 2019a, 2019b; Leclerc et al., 2009). Third, we restricted our selection to cases in which the offender and their victim were strangers at the time of the rape (i.e., they had no relationship prior to the crime). We made this choice to have a more homogeneous sample. Previous studies showed different offending patterns in cases of rape committed against strangers as opposed to acquaintances (see Bownes et al., 1991; Chopin & Beauregard, 2020b; Koss et al., 1988). Moreover, individuals involved in acquaintance rapes may have less of a need to use forensic awareness strategies because they are known by their victim and can be easily identified (Chopin et al., 2019). Finally, we decided to exclude rape cases with a lethal outcome (i.e., sexual homicides) because several studies found that they present several difference in terms of crime-commission process and offender characteristics (see Beauregard & DeLisi, 2018a, 2018b; Beauregard et al., 2018, 2020; Chopin & Beauregard, 2019a, 2019c).

#### Research subjects

Victims included in the sample are all women, who were on average 31.31 (*SD* = 16.41, range = 16–82) years old. Most victims were in a relationship at the time of the crime (70.6%; *n* = 1,095) and they were assaulted while walking/jogging (52.7%; *n* = 818), while involved in domestic activities (10.3%; *n* = 159), while drinking at a bar (8.1%; *n* = 125), or while involved in prostitution (8%; *n* = 124). Victims were 16.8% of victims to use alcohol (*n* = 260) and 4.3% to use drugs (*n* = 66) at the time of the offense. Approximately one quarter of victims (22.2%; *n* = 345) had an active social life (i.e., partying, participates in social situations and attends events where other people, including acquaintances and strangers, gather), whereas few of them (2.8%; *n* = 44) had a loner lifestyle (i.e., victims have a loner lifestyle with few social interactions).

Offenders were all men, aged on average of 29.52 (*SD* = 9.17, range = 14–70) years old. Similar to the victims’ profiles, they were mostly in a relationship at the time of the crime (61.9%; *n* = 960) and approximately one quarter (24.2%; *n* = 376) of offenders had consumed alcohol prior to the offenses, whereas 15.1% (*n* = 234) of them had used drugs. A small proportion of offenders had a previous criminal history^1^ (14.8%; *n* = 230), whereas 13.1% (*n* = 203) of them were characterized by some paraphilic behaviors (i.e., type of behavior associated with any paraphilias but without having to meet the diagnostic criteria, see Chopin, Beauregard, Gatherias, & Oliveira, 2020). Few offenders (5.7%; *n* = 88) lead an active social life (i.e., partying, participates in social situations and attends events where other people, including acquaintances and strangers, gather) or lead a loner lifestyle (5.9%; *n* = 93; that is, victims have a loner lifestyle with few social interactions).

### Measures

#### Dependent variables

Three dependent variables were included in this study. These dependent variables were computed based on 24 dichotomous variables describing forensic awareness strategies used by individuals who have committed stranger rapes at the time the offense (see Table 1). The first dependent variable is dichotomous and measures whether at least one forensic awareness strategy was used by the offender, with 0 indicating the absence of a forensic awareness strategy (*n* = 688) and 1 indicating at least one forensic awareness strategy was used (*n* = 863). The second dependent variable is continuous and measures the number of forensic awareness strategies used by each offender, ranging from 0 to 9 strategies used (*M* = 1.18, *SD* = 1.53). This variable was nonnormally distributed, with skewness of 1.78 (*SE* = 0.06) and kurtosis of 3.74 (*SE* = 0.12).

**Table 1.** Descriptive Analysis of Forensic Awareness Strategies Used by Individuals Who Have Committed Stranger Rapes (*N* = 1,551).

Type of forensic awareness strategies	*n*	% of total sample (*N* = 1,551)	% of forensic awareness strategies users (*n* = 863)
Protecting identity			
Offender covered mouth/gagged victim	331	21.34	38.35
Offender told/threatened/bribed victim to not report	307	19.79	35.57
Offender used a condom	183	11.80	21.21
Offender disabled telephone	118	7.61	13.67
Offender wore mask	114	7.35	13.21
Offender covered victim’s eyes or face	93	6.00	10.78
Offender gave a false name	87	5.61	10.08
Offender bound victim	86	5.54	9.97
Offender wore gloves	81	5.22	9.39
Offender closed door(s) and/or window(s)	45	2.90	5.21
Offender disabled/darkened lighting	35	2.26	4.06
Offender administrated drug/alcohol/substance to victim	30	1.93	3.48
Offender wore dark/extra/concealing clothing	14	0.90	1.62
Offender used a lookout	13	0.84	1.51
Offender disguised/changed physical appearance	3	0.19	0.35
Offender wore disguise	2	0.13	0.23
Offender used an alarm system	1	0.06	0.12
Offender relocated postcrime	1	0.06	0.12
Destroying evidence			
Offender removed or destroyed forensic evidence	116	7.48	13.44
Offender forced victim to bathe or douche	36	2.32	4.17
Offender cleared scene	27	1.74	3.13
Offender planted evidence/staged scene	7	0.45	0.81
Offender set fire to scene	6	0.39	0.70
No. of forensic awareness strategies			
0	688	44.36	
1	395	25.47	45.77
2	223	14.38	25.84
3	118	7.61	13.67
4	64	4.13	7.42
5	26	1.68	3.01
6	17	1.10	1.97
7	11	0.71	1.27
8	7	0.45	0.81
9	2	0.13	0.23

The third dependent variable is categorical and measures the type of strategies used by the offenders. Three categories of forensic awareness strategies were created: absence of forensic awareness strategies (coded 0), forensic awareness strategies used to protect the offender’s identity (coded 1), and forensic awareness strategies used to destroy evidence (coded 2). This last category is considered as the most sophisticated type of forensic awareness strategies (see Beauregard & Bouchard, 2010; Chopin, Beauregard, & Bitzer, 2020; Chopin et al., 2019). Cases in which offenders used both strategies to protect their identity and to destroy forensic evidences are included in the third category.

#### Independent variables

As was discussed previously, criminal expertise can be partly operationalized using the ability of sex offenders to use a sophisticated modus operandi. A sophisticated modus operandi can be divided in several steps to describe the various actions taken to successfully complete the crime (Beauregard & Proulx, 2017). Based on previous studies, we used 10 variables divided under three main subcategories (i.e., planning the crime, locations chosen by the offenders, rewarding behavior during the crime) to measure the sophistication of modus operandi. These variables were chosen because they capture the ability of sex offenders to maximize the benefits associated with the crime and reduce the risks associated with police detection. It is important to mention here that with the exception of one variable (i.e., number of sexual acts committed), all the independent variables used in this study were originally coded by a team of crime analysts from criminal investigation files and forensic expertise reports. As such, the coding scheme originally determined by the team of crime analysis was used in this study, we did not recode any of the variables.

##### Planning the crime

Previous studies have shown that expert offenders are more likely to plan and anticipate their crime (Beauregard & Proulx, 2017; Nee & Meenaghan, 2006). We used two dichotomous variables to examine this dimension: (a) victim targeted by offenders (0 = absence;1 = presence), and (b) strategy of approach: con (e.g., befriended the victim, posed as an authority figure, offered assistance; 0 = absence; 1 = presence).

##### Locations chosen by the offenders

Previous studies have found that sophisticated offenders were more likely to select crime locations characterized as being lower risk (Beauregard & Proulx, 2017; Ceccato, 2014; Chopin & Caneppele, 2019b; Nee & Meenaghan, 2006). We used four dichotomous variables to test this assumption: (c) deserted crime location (as opposed to locations where witnesses can hear, see, and interrupt the crime; 0 = absence, 1 = presence), (d) indoor crime location (0 = absence, 1 = presence), (e) familiar crime location (0 = absence, 1 = presence), and (f) offender uses single crime location (0 = absence, 1 = presence).

##### Rewarding behaviors during the crime phase

Previous studies assert that the ability to maximize the payoffs for sexual offenders can be assessed with the number of sexual contacts perpetrated (Lussier et al., 2011). Moreover, Beauregard and Proulx (2017) suggested that sophisticated sexual offenders are able to control the crime commission by bringing a weapon to frighten the victim and avoid victim resistance. Accordingly, the ability of an offender to maintain control during the entire crime commission process qualifies as an element of criminal expertise.

We used one continuous and three dichotomous variables to test these aspects: (g) number of sexual acts committed (*M* = 2.35, *SD* = 1.18, range = 1–6); this continuous variable was computed from the following sexual acts: vaginal penetration with a penis, anal penetration with a penis, fellatio, masturbation, foreign object insertion (vaginal and/or anal), and cunnilingus. As only rape cases were considered for this study, at least one act of vaginal/anal penetration was present per case: (h) absence of physical resistance from the victim (0 = absence, 1 = presence), (i) weapon is brought at the crime scene to threaten the victim (0 = absence, 1 = presence), and (j) whether offenders intentionally released their victims (i.e., reflects a better control than when the victim ran away or was released by a third party; 0 = absence; 1 = presence).

### Analytical Strategy

As our goal was to identify whether offenders whose modus operandi is indicative of having criminal expertise are more likely to use forensic awareness strategies, data were analyzed following a two-step process. The first analytical step consisted in a bivariate comparison of the modus operandi characteristics of individuals who have committed stranger rapes who used forensic awareness strategies and those who did not. The second analytical step included three multivariate analyses to explore different subcategories of skills used by offenders to avoid police detection. Specifically, we tested whether different types and number of forensic awareness strategies used by sexual offenders were associated with sophisticated modus operandi variables. As suggested by several references in statistics (see Hosmer & Lemeshow, 2013; Tabachnick & Fidell, 2019), it is not recommended to perform multivariate analyses when bivariate analyses indicate that there is no relationship between two variables. In the case where a significant relationship is observed at the multivariate level despite the absence of a significant relationship at the bivariate level, it is likely to be due to interaction effects with other variables. Consequently, first we used only variables that were significant at the bivariate level. Thereafter, a binomial logit regression model was used to identify variables that characterize the use of forensic awareness strategies. Second, using the same independent variables, we conducted a negative binomial regression^2^ to examine whether the number of forensic awareness strategies used by the offenders was influenced by a modus operandi indicative of expertise. Finally, we computed a multinomial regression to compare the expert modus operandi of offenders destroying forensic evidence with offenders who protect their identity as well as those who do not use forensic awareness strategies. Test for multicollinearity was conducted for the variables included in the multivariate analyses, and results show that the variance inflation factor (VIF) static did not exceed the 1.045 threshold and the tolerance was above 0.989. A power analysis was also conducted to determine the appropriateness of our sample size, and results reveal that the observed power values were above 0.82 and statistically significant at the *p* ≤ .05 level.

We report how we determined our sample size, all data exclusions, all manipulations, and all measures in the study. Research ethics approval was not required for this study.

## Results

Table 1 provides an overview of the forensic awareness strategies used by individuals who have committed stranger rapes. The most frequent forensic awareness strategies aiming at protecting the stranger rapists’ identity are covering the victim’s mouth (21.34%), threatening the victim not to report the crime (19.79%), using a condom (11.80%), disabling victims’ telephones (7.61%), and wearing a mask (7.35%). As to modus operandi of destroying evidence, forensic awareness strategies most frequently used were removing or destroying forensic evidence (7.48%), forcing victims to shower (2.32%), and cleaning the scene after the crime (1.74%). These findings also show that more than half of the individuals who have committed stranger rapes used at least one forensic awareness strategy (55.64%). Among these forensically aware offenders (*n* = 863), 45.77% used only one strategy, whereas 46.93% used between two and four strategies and 7.30% used between five and nine strategies.

Table 2 presents results of the bivariate analyses. Rapists using forensic awareness strategies adopted a con approach less frequently (Cramer’s *V* = .160, *p* < .001), but more often targeted their victim (Cramer’s *V* = .112, *p* < .001) as well as assaulted them in a deserted (Cramer’s *V* = .080, *p* = .002) and indoor location (Cramer’s *V* = .067, *p* = .009). During the assaults, individuals who have committed stranger rapes using forensic awareness strategies perpetrated more sexual acts (*M* = 2.53) compared with nonforensically aware stranger rapists (*M* = 2.35; *U* = 239,348, *p* = .001, *r* = .16). Victims assaulted by individuals who have committed stranger rapes using forensic awareness strategies were less likely to physically resist (Cramer’s *V* = .112, *p* < .001), whereas stranger rapists using forensic awareness strategies brought a weapon with them less often (Cramer’s *V* = .063, *p* = .014). Finally, when a forensic awareness strategy was used, victims were more often intentionally released (Cramer’s *V* = .173, *p* < .001).

**Table 2.** Bivariate Analysis Between the Use of Forensic Awareness and the Modus Operandi Characteristics (*N* = 1,551).

	Total		No forensic awareness		Forensic awareness		Cramer’s *V*/φ Mann–Whitney *U* test	*p*
	*N* = 1,551		*n* = 688		*n* = 863			
Variables	*n*	%	*n*	%	*n*	%		
Victim was targeted by offender	284	18.31	92	13.37	192	22.25	.112***	.000
Strategy of approach: Con	858	55.32	442	64.24	416	48.20	.160***	.000
Deserted place	914	58.93	375	54.51	539	62.46	.080***	.002
Inside place	795	51.26	327	47.53	468	54.23	.067**	.009
Offender is familiar with the place of crime	563	36.30	258	37.50	305	35.34	.022	.380
Offender uses single crime location	675	43.52	295	42.88	380	44.03	.021	.650
Number of sexual acts committed	2.35 (1–6)		2.14 (1–6)		2.53 (1–6)		239,348***	.000
Victim did not physically resist	836	53.90	328	47.67	508	58.86	.112***	.000
Presence of weapon	319	20.57	161	23.40	158	18.31	.063*	.014
Victim was intentionally release	1,087	70.08	421	61.19	666	77.17	.173***	.000

* *p* < .05. **p ≤ .01. ***p ≤ .001.

Table 3 presents findings of the binomial regression testing differences in the crime process of individuals who have committed stranger rapes using or not using forensic awareness strategies. Results show that individuals who have committed stranger rapes using forensic awareness strategies were more likely to target their victims (β = .525, *p* < .001), but less likely to use a con approach (β = −.640, *p* < .001). They were also more likely to choose a deserted (β = .241, *p* = .029) and indoor crime (β *=* .199, *p* = .035) location. Individuals who have committed stranger rapes using forensic awareness strategies were more likely to perpetrate a greater number of sexual acts during the criminal event (β = .258, *p* < .001) and to intentionally release their victims (β = .690, *p* < .001). However, individuals who have committed stranger rapes using forensic awareness strategies were less likely to bring a weapon with them (β = *−*.255, *p* < .001).

**Table 3.** Binomial Regression Analysis on the Use of Forensic Awareness and the Modus Operandi Characteristics (*N* = 1,551).

Variables	β	*SE*	*p*-value
Victim was targeted by offender	.525***	.147	.000
Strategy of approach: Con	−.64***	.109	.000
Deserted place	.241*	.110	.029
Inside place	.199*	.108	.380
Number of sexual acts committed	.258***	.047	.000
Victim did not physically resist	.167	.157	.287
Presence of weapon	−.255*	.134	.046
Victim was intentionally release	.690***	.117	.000
Constant	−.690***	.176	.000
χ^2^	151.989***		
−2 log likelihood	1,978.366		
Hosmer–Lemeshow test	8.985		

* *p* < .05. ***p ≤ .001.

Table 4 reports findings of the negative binomial regression testing differences in the crime process of individuals who have committed stranger rapes based on the number of forensic awareness strategies used. Results show that individuals who have committed stranger rapes were more likely to use several forensic awareness strategies when they also targeted their victims (β = .276, *p* = .002), but did not use a con approach (β = −.438, *p* < .001). In addition, individuals who have committed stranger rapes were more likely to use several forensic awareness strategies when they committed the crime indoor (β = .353, *p* < .001), when a greater number of sexual acts have been perpetrated (β = .160, *p* < .001), when victims did not physically resist (β = .240, *p* < .010), and when they were intentionally released (β = .160, *p* < .001).

**Table 4.** Negative Binomial Regression Analysis on the Number of Forensic Awareness Used and the Modus Operandi Characteristics (*N* = 1,551).

Variables	β	*SE*	95% Wald confidence interval	*p*
Victim was targeted by offender	.276**	.0890	[.101, .450]	.002
Strategy of approach: Con	−.438***	.0714	[−.578, −.298]	.000
Deserted place	.104	.0733	[−.040, .247]	.156
Inside place	.353***	.0720	[.212, .494]	.000
Number of sexual acts committed	.160***	.0303	[.100, .219]	.000
Victim did not physically resist	.240**	.0937	[.056, .424]	.010
Presence of weapon	−.143	.0916	[−.323, .036]	.117
Victim was intentionally release	.160***	.0815	[.186, .506]	.000
Constant	−.612***	.1234	[−.854, −.370]	.000
Likelihood ratio χ^2^	171.887***			
AIC	4,515.031			
AICc	4,515.148			
BIC	4,563.151			
Deviance	1,337.650			

*Note.* AIC = Akaike information criterion; BIC = Bayesian information criterion; AICc = Akaike information criterion corrected.

**p ≤ .01. ***p ≤ .001.

Table 5 presents the results of the multinomial regression for which the reference category variable was the destruction of forensic evidence. Model 1 compared the modus operandi characteristics of stranger rapes during which offenders used strategies to destroy evidence and rapes during which offenders did not use forensic awareness strategies. Findings of Model 1 suggest that there are several differences between the two categories. When individuals who have committed stranger rapes used strategies to destroy forensic evidence, they less often used a con approach (β = −.777, *p* < .001). Individuals who have committed stranger rapes using no forensic awareness strategies perpetrated a smaller number of different sexual acts (β = −.302, *p* = .000). Offenders who destroyed forensic evidences more frequently committed their crime in an indoor place (β = .932, *p* < .001). In addition, their victims were more likely to show no physical resistance (β = .520, *p* = .025), and they were more often intentionally released (β = .456, *p* = .001).

**Table 5.** Multinomial Regression Results With Type of Forensic Awareness as Dependent Variable and Destruction of Evidence as Reference Category (*N* = 1,551).

	Model 1^a^			Model 2^b^		
Variables	β	*SE*	*p*	β	*SE*	*p*
Victim was targeted by offender	.455	.232	.065	−.068	.220	.758
Strategy of approach: Con	−.777***	.187	.000	−.193	.184	.294
Deserted place	.354	.194	.069	.166	.192	.386
Inside place	.932***	.199	.000	.851***	.197	.000
Number of sexual acts committed	−.302***	.078	.000	−.052	.075	.950
Victim did not physically resist	.520*	.233	.025	.460	.221	.068
Presence of weapon	−.307	.243	.906	−.098	.242	.685
Victim was intentionally release	.456*	.210	.030	−.221	.212	.298
Constant	1.391***	.425	.001	1.198**	.414	.004
−2 log likelihood	1,394.465					
AIC	1,258.462					
BIC	1,354.702					
χ^2^	172.003***					

*Note.* AIC = Akaike information criterion; BIC = Bayesian information criterion.

a Destruction of evidence (reference category) versus no forensic awareness strategy. ^b^Destruction of evidence (reference category) versus protecting identity.

* *p* < .05. **p ≤ .01. ***p ≤ .001.

Model 2 compared the modus operandi characteristics of stranger rapes during which offenders used strategies to destroy forensic evidence and rapes during which offenders used only strategies to protect their identify. Results indicate only one significant difference concerning the crime location. Cases in which individuals who have committed stranger rapes destroyed forensic evidences occurred more often at an indoor location in comparison with cases in which stranger rapists only protected their identity (β = .851, *p* < .001).

## Discussion

Our findings suggest that overall, individuals who have committed stranger rapes who adopt behaviors indicative of expertise are also more likely to use forensic awareness strategies with the purpose of decreasing the risk of being detected by police. Looking at the early stage of the crime-commission process, our findings show that forensically aware offenders are also more likely to target their victims prior to the crime. As suggested by Beauregard and Proulx (2017), a sophisticated modus operandi is usually well prepared and victim selection is a feature of crime anticipation and preparation. While preparing for the commission of their crime, offenders have time to anticipate and assess the perceived risks. This allows them to make better decisions as to which strategies should be used to increase their chances of avoiding police detection. Surprisingly though, a noncoercive approach—which has been traditionally related to a sophisticated modus operandi (i.e., lack of violence; see Beauregard & Proulx, 2017)—was strongly associated with the absence of forensic awareness strategies. This can be interpreted different ways. First, as suggested by Beauregard and Bouchard (2010), it can be that offenders using a noncoercive approach assumed the victim’s consent or that no forensic traces would be left on the scene due to the absence of physical violence involved during the crime. Second, it is possible that a noncoercive and a con approach are not a feature of expertise in specific cases of stranger rape. As all victims from this study were complete strangers, this would mean that noncoercive approach methods are ineffective as most offenders must achieve control and limit victims’ reactions (e.g., physical resistance, victims’ cries) by using a coercive and surprise approach. This adaptation of the strategies used to commit the crime to the specific context (i.e., stranger rape) is indicative of rational decision-making and hence expertise.

Another aspect related to the expertise in sexual crimes concerns the chosen locations to commit the crime. Cases in which offenders chose deserted and indoor locations were strongly associated with the use of forensic awareness strategies to avoid police detection. Rapists assaulting their victims at deserted locations limit the risks of being identified and interrupted by witnesses (Ceccato, 2014). In such situations, offenders have more time during the crime to use forensic awareness strategies to avoid police detection. The reasoning appears similar for cases occurring indoor. Offenders limit the risks to be seen or interrupted during their assault. However, Chopin et al. (2019) found that sexual crimes committed outdoors are more difficult to solve by the police, mainly because forensic evidence in these cases are more difficult to retrieve by investigators (Martin et al., 2019). Therefore, the decision regarding the chosen locations to commit the crime represents an interesting trade-off for the offender. It can be hypothesized that depending on the most important benefit pursued by the offender—whether it is to avoid detection or to obtain sexual gratification—the offender may choose a different crime location based on what will provide protection against witnesses or enable evidence destruction. Such decision-making illustrates perfectly the bounded rationality (see Gigerenzer & Selten, 2002) and the importance to consider the expertise in the specific context of the crime (Nee & Ward, 2015).

One way to examine this “dysfunctional” expertise in sexual crimes is to consider the level of sexual intercourse achieved during the criminal event. Expert sexual offenders could be considered as those able to achieve the greatest level of sexual intercourse with the victim while simultaneously limiting the risks associated with their behaviors (Lussier et al., 2011). Our findings give support to this idea that individuals who have committed stranger rapes and perpetrated a greater number of sexual acts were also more likely to use forensic awareness strategies to avoid police detection. Here, the association between the higher level of expertise in the modus operandi and the use of forensic awareness strategies may be partly explained by the Locard’s (1920) exchange principle. The greater the number or the more intrusive (i.e., penetration) the sexual acts, the greater the probability to leave forensic evidence at the crime scene. Therefore, for expert offenders who penetrate their victim or commit multiple sexual acts, it becomes important to adopt forensic awareness strategies.

### More Expertise, Different Forensic Awareness Strategies?

Our study tested whether having expertise in the modus operandi used during rape would predict the use of forensic awareness strategies to avoid police detection. Looking at the extent of strategies employed by offenders, findings suggest that offenders who use a greater number of forensic awareness strategies and demonstrate having the expertise to avoid police detection (i.e., protecting identity vs. destroying evidence) were also strongly influenced by the expertise of the modus operandi. Thus, the more expertise exhibited in their modus operandi, the more individuals who have committed stranger rapes use various forensic awareness strategies and possibly thwart the police investigation.

Further exploration into the categories of forensic awareness strategies revealed significant differences between individuals who committed stranger rapes and destroyed forensic evidence and those who did not use forensic awareness strategies. As expected, findings conformed to the same trend as the one found in the analyses comparing cases with and without forensic awareness strategies. However, the comparative analysis among individuals who have committed stranger rapes who did use forensic awareness strategies revealed very few differences between the types of forensic awareness strategies used (i.e., destroying forensic evidence vs. protecting identity). The only significant difference relates to the location where the offense took place. More specifically, rapes occurring indoors were more frequently related to offenders who destroyed forensic evidence as opposed to solely protecting their identity. This finding suggests that the use of forensic awareness strategies aimed at destroying evidence is driven by the crime context and the offender having awareness of the ability of forensic identification services to better trace and detect evidence indoors (see Chopin et al., 2019; Martin et al., 2019). We cannot, however, exclude that an individual destroying evidence during an indoor rape would not use the same forensic awareness strategies outdoors. The type of forensic awareness strategies used by individuals who have committed stranger rapes is perhaps more reflective of an adaptation to the context of the crime than a different—or higher—level of expertise.

### What Is Expertise in Sexual Offending?

Although no comprehensive definition of expertise in sexual offending exists, Beauregard and Proulx (2017) suggested that well-planned crimes present sophistication, whereas Park et al. (2008) focused on the various decisions taken to avoid police detection as an indicator of expertise. Ward (1999) suggested that avoiding police detection over many years with several victims was the result of a specific expertise, such as taking precautions with offense locations, being able to regulate their emotional state, deceiving people close to them, and conducting constant risk appraisal that enable them to avoid detection. Such strategies could then be incorporated in multiple scripts about different elements of an offense, increasing the chances for an offender to successfully complete the crime and avoid police detection. Combining these various aspects related to the crime, we postulate that the expert sexual offender should demonstrate a strong level of crime planning, controlling its process from the precrime phase to the end of the crime, being able to perform varied and intrusive sexual acts while also adopting forensic awareness strategies. The expert stranger rapist is an individual involved in a rape who can combine a sophisticated modus operandi and the use of forensic awareness strategies.

### Toward a Theoretical Classification of Criminal Expertise in Sexual Offending

As suggested by the RCT (Cornish & Clarke, 1986, 1987), to maximize the payoffs associated with the crime, some offenders will adopt behavioral strategies indicative of expertise to keep their crime under control, limit the risks to be arrested, and maximize the benefits of their assaults (e.g., sexual gratification, domination, thrill). To reduce the risks associated with police detection, some offenders use forensic awareness strategies (Davies, 1992). The current study provides empirical evidence to support RCT as only half of participants employed forensic awareness strategies. The use of forensic awareness strategies was also associated with a sophisticated modus operandi results and therefore provides some support that sexual offenders have a certain degree of expertise when committing rape. The combination of both the modus operandi and the use of forensic awareness strategies helps inform four-type classification of expertise (see Table 6). This theoretical classification was generated using the four possibilities resulting from the combination of the use—or not—of a sophisticated modus operandi and the use—or not—of forensic awareness strategies. The first profile was labeled the *novice stranger rapists* as it described offenders who present a basic modus operandi and an absence of forensic awareness strategies. These offenders are not aware of what strategies work best when assaulting a victim (e.g., avoiding physical resistance, avoiding witnesses’ interference) and do not appear to understand what needs to be done to remove evidence from the crime scene to aid in detection avoidance. The second profile was labeled the *bold stranger rapists* as it described offenders who displayed a sophisticated modus operandi without using any forensic awareness strategies. These offenders have adopted a modus operandi that allowed them to sexually assault the victim, whereas the use of extra strategies to avoid detection (i.e., forensic awareness strategies) did not seem necessary. The third profile was labeled the *opportunistic stranger rapists* as it described offenders who despite using a basic modus operandi still adopt forensic awareness strategies. These offenders typically encounter a crime opportunity without planning and react during or after the crime by adopting forensic awareness strategies. Finally, the fourth profile was labeled *expert stranger rapists* as it described offenders with the highest level of expertise in rape. These offenders use both a sophisticated modus operandi and forensic awareness strategies. They know that to maximize their chances of avoiding detection, they need to use a sophisticated modus operandi paired with specific forensic awareness strategies that increase the odds of eluding police detection. This theoretical classification proposes new dimensions to the typology proposed by Bourke et al. (2012) in which they propose to identify expertise in child abuse across a continuum. Beyond the expert and novice categories described by Bourke et al. (2012), we assume the presence of two other categories of offenders based on the presence/absence of either a sophisticated modus operandi or the use of detection avoidance strategies.

**Table 6.** Theoretical Classification of Rapists According to Their Level of Expertise.

	No forensic awareness strategies	Forensic awareness strategies
Basic modus operandi	Novice rapist	Opportunistic rapist
Sophisticated modus operandi	Bold rapist	Expert rapist

## Limitations and Future Directions

Evaluation into the role of expertise in the context of sexual offending is instructive. However, it is important that we acknowledge study limitations. First, cases included in this study were all considered as nonserial rapes. As mentioned by Chopin and Aebi (2018), in some situations police failed to identify links between cases. This leads to the question of whether individuals who have committed serial stranger rapes—due to their repeated experience with a specific type of crime—present a more elaborated expertise that allow them to avoid police detection for longer? Future studies should attempt to replicate our findings with serial cases to specifically test the role that experience and expertise play on the type of strategies used by sexual offenders to avoid police detection. Second, this study was based on police data and present some methodological biases in terms of validity and reliability (see, for example, Aebi, 2006; Chopin & Aebi, 2018, 2019). Third, we only used nine variables to describe the modus operandi. As mentioned by Chopin and Beauregard (2020b), the use of quantitative data to describe the crime-commission process presents a number of benefits but lead to a simplification of a complex set of interactions constituting the criminal event. More information, especially concerning the offender–victim interactions, would be interesting to analyze, to increase our understanding of the concept of criminal expertise in sexual crimes. Fourth, in this study, we included only nonserial stranger rape cases in the sample and findings cannot be generalized to individuals who have committed serial stranger rapes. Fifth, because these data consist only of solved cases, there exists the possibility for selection bias. By definition, unsolved cases were not included and, therefore, the most “expert” stranger rapist may have been excluded in our analysis, and having expertise is associated with a lower likelihood of detection and, hence, a greater likelihood of the crime being unsolved. Finally, we used a sample of offenders with limited criminal experience. For this reason, we are aware that using data that include a sample of offenders with prior criminal convictions may lead to a different set of results with regard to criminal expertise. However, it is important to keep in mind that expertise is not only acquired through previous convictions. Some offenders do not get identified and/or arrested for many of the crimes they commit because they have developed an expertise to avoid detection. In addition, as aforementioned, for some offenders, the development of expertise is obtained through other means (e.g., observation, sexual fantasies).

Conclusions of this study should be applied only to police-reported cases, which constitute only a minority of rape committed (Chopin, 2017). Cross validation of the current findings with nonpolice samples (e.g., interviews conducting with victims of rape and individuals involved in rape cases themselves) could provide further information on the behaviors adopted by offenders during rape. Further studies should use statistical technics such as latent class analysis to test the theoretical classification we propose and identify the various classes of offenders according to their abilities to follow a sophisticated modus operandi and to use detection avoidance strategies. The concept of having criminal expertise should also be studied using different methodological approaches—for instance, with the use of an experimental process with staged scenarios or virtual reality to provide more concrete findings.

## Conclusion

This study was the first to empirically focus on the criminal expertise of men who sexually offend against adult women. The concept of expertise in offending may be tied to the rational choice approach, as it assumes that sexual offenders are driven by two main abilities: maximizing the payoffs and minimizing negative consequences, such as being detected by the police. Our findings showed that individuals who have committed stranger rapes and who demonstrated expertise in their modus operandi were also more likely to use forensic awareness strategies. Also, our results showed a positive relationship between the modus operandi expertise and the number of forensic awareness strategies used by individuals who have committed stranger rapes, but there was no clear association between the modus operandi expertise and the types of forensic awareness strategies (destroying evidence vs. protecting identity). We believe that the number of forensic awareness strategies used by an individual involved in a stranger rape case is an indicator of his expertise, whereas the type of forensic awareness strategies represents an adaptation of the situational constraints related to the crime.

Findings from this study have several theoretical and practical implications. From a theoretical perspective, this study confirmed that individuals who have committed stranger rapes and who developed a certain criminal expertise in the modus operandi are also more likely to have developed an expertise associated with a greater likelihood of avoiding police detection (i.e., forensic awareness strategies). Moreover, the study showed that although sexual offenders may be capable of rational decision-making (see, for example, Beauregard & Leclerc, 2007) and influenced by both internal and external constraints, they do not share the same skills whenever committing crimes. Also, the finding that some nonserial stranger rapists may demonstrate criminal expertise suggests that the learning process involved in the development of expertise is not only limited to the repetition of the behavior but also to some modeling (see Ward, 1999). Moreover, it is important to mention here that even with a certain level of expertise, these individuals who have committed stranger rapes have been detected by the police. This suggests that the detection of these offenders does not depend solely on their abilities and skills. This is congruent with the notion of bounded rationality (Simon, 1957; see also Gigerenzer & Selten, 2002) where offenders may use heuristics, simple decisions, with incomplete information. This is further complicated by the fact that depending on the main objective—or payoff—pursued by the offender, the decisions and strategies used during the crime may decrease certain risks while increasing others. Factors such as cognitive biases (Dror, 2011), errors in judgment (Chi, 2006), risky decision-making (Weinborn et al., 2013), and even the offender’s affect prior to the crime (Van Gelder, 2013) may all impact the decision-making process during the commission of the crime.

As to practical implications, the concept of expertise may be useful for treatment as sexual offenders are often seen as novices by professionals (see Ciardha, 2015). As suggested by Nee and Meenaghan (2006), such expertise may translate into “action slip” (see Sellen & Norman, 1992) where offenders who are stressed out by cognitive overload or emotional events (e.g., relationship problems, difficulties finding a place to live after being released) may act out some of these automatic, overlearned behaviors. Therefore, it becomes important for the various actors involved in the follow-up of the offender to be aware of these potential slips that could lead to recidivism. Finally, as some sexual offenders may be more difficult to treat due to their accumulated “expertise,” information on the presence and level of expertise involved in the crime-commission process may help identify some of the cognitive mechanisms used by these offenders to delay or block interventions as well as those that may help (see Bourke et al., 2012). Thus, knowledge gained from the current study could benefit practitioner in providing more adapted intervention.

## Notes

1 Specific details on previous crime committed are not available.

2 Negative binomial regression has been preferred to multiple regression because the dependent variable did not follow a normal distribution.
